# Molecular Mimics of Classic P-Glycoprotein Inhibitors as Multidrug Resistance Suppressors and Their Synergistic Effect on Paclitaxel

**DOI:** 10.1371/journal.pone.0168938

**Published:** 2017-01-09

**Authors:** Moustafa E. El-Araby, Abdelsattar M. Omar, Maan T. Khayat, Hanan A. Assiri, Ahmed M. Al-Abd

**Affiliations:** 1 Department of Pharmaceutical Chemistry, Faculty of Pharmacy, King Abdulaziz University, Jeddah, Saudi Arabia; 2 Department of Pharmaceutical Organic Chemistry, Faculty of Pharmacy, Helwan University, Cairo, Egypt; 3 Department of Pharmaceutical Chemistry, Faculty of Pharmacy, Al-Azhar University, Cairo, Egypt; 4 Department of Biochemistry, Faculty of Science, King Abdulaziz University, Jeddah, Saudi Arabia; 5 Department of Pharmacology and Toxicology, Faculty of Pharmacy, King Abdulaziz University, Jeddah, Saudi Arabia; 6 Department of Pharmacology, Medical Division, National Research Centre, Cairo, Egypt; Ain Shams University, EGYPT

## Abstract

P-glycoprotein (Pgp) is a membrane bound efflux pump spread in a variety of tumor cells and considered as a main component of multidrug resistance (MDR) to chemotherapies. In this work, three groups of compounds (imidazolone, oxazolone and vinyl dipeptide derivatives) were synthesized aiming to develop a molecular framework that effectively suppresses MDR. When tested for their influence on Pgp activity, four compounds coded Cur1-01, Cur1-12V, Curox-1 and Curox-3 significantly decreased remaining ATP concentration indicating Pgp substrate site blocking. On the other hand, Cur-3 and Cur-10 significantly increased remaining ATP concentration, which is indicative of Pgp ATPase inhibition. The cytotoxicity of synthesized compounds was examined against Pgp expressing/highly resistant colorectal cancer cell lines (LS-174T). Compounds Cur-1 and Cur-3 showed considerable cytotoxicity with IC_50_ values of 7.6 and 8.9 μM, respectively. Equitoxic combination (at IC_50_ concentrations) of PTX and Cur-3 greatly diminished resistant cell clone from 45.7% to 2.5%, albeit with some drop in potency from IC_50_ of 7.9 nM to IC_50_ of 23.8 nM. On the other hand, combination of PTX and the non-cytotoxic Cur1-12V (10 μM) significantly decreased the IC_50_ of PTX to 3.8 nM as well as the resistant fraction to 16.2%. The combination test was confirmed using the same protocol but on another resistant CRC cell line (HCT-116) as we obtained similar results. Both Cur-3 and Cur1-12V (10 μM) significantly increased the cellular entrapment of Pgp probe (doxorubicin) elevating its intracellular concentration from 1.9 pmole/cell to 3.0 and 2.9 pmole/cell, respectively.

## Introduction

Resistance to chemotherapy has been standing as the major obstacle to declare a clear victory in the fight with the “Emperor of All Maladies” [1, 2]. There are several mechanisms by which tumor cells develop resistance towards cytotoxic drugs aiming to stay alive and continue their malignant pattern of growth and proliferation [3, 4]. Multidrug resistance through efflux of chemotherapeutic agents outside cancer cells is recognized as a major cancer resistance mechanism that is responsible for failure of treatment after initial rapid improvement [5]. When patients are placed in a chemotherapy regimen, cancer cells strike back by over expression of ATP-dependent Binding Cassette (ABC) transporter proteins. ABC is a superfamily of membrane-bound transporters that are able to uptake xenobiotics and chemical substances unidirectional from inside to outside the cell [6]. The earliest discovered and most studied ABC transporter protein is the P-glycoprotein (Pgp); it is also known as multidrug resistance protein 1 (MDR-1), ATP-binding cassette sub-family B member 1 (ABCB1) or cluster of differentiation 243 (CD243). Pgp was first identified by Victor Ling and coworkers in 1970s as a protein responsible for multidrug permeability in Chinese hamster ovary cells [7, 8]. After cloning of Pgp cDNA [9], its wild gene alleles (as well as mutant gene alleles) were found to be amplified in tumor cells as a response to chemotherapeutic agents leading to development and spread of MDR events within tumor cells [10].

**Fig 1:**
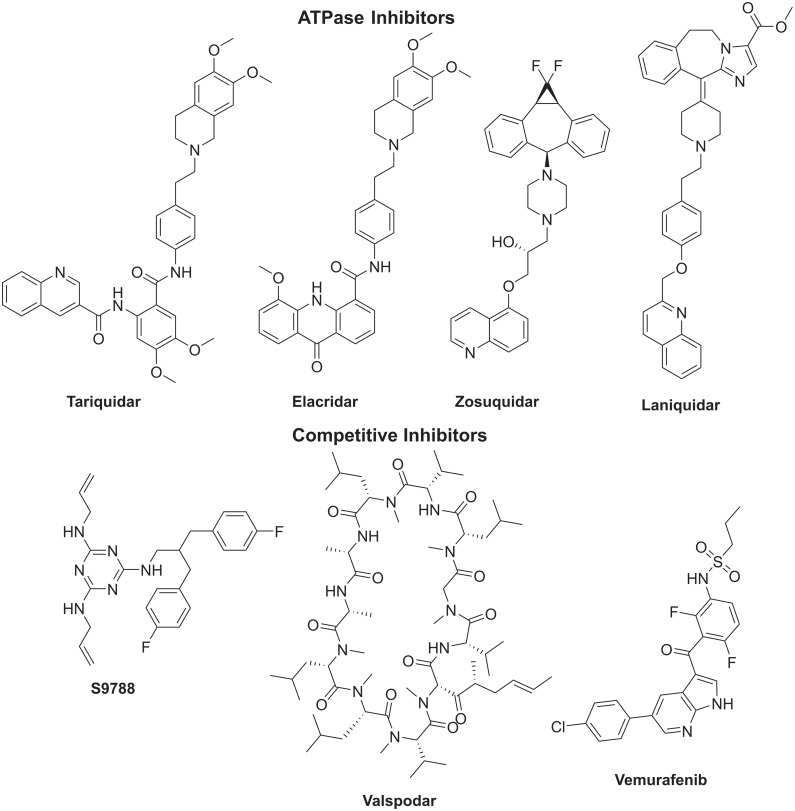
Examples of Pgp inhibitors.

The X-ray crystal structure of mouse Pgp protein (87% similarity to human Pgp), resolved in 2009, was described as two trans-membrane domains each composed of six α-helices opening inward during the relaxed state [11]. Each domain is linked in its intracellular face to a nucleotide-binding domain (NBD) which is also known as the ATPase motif of the pump. The surface between the two leaflets is wide and largely hydrophobic. Crystallographic data as well as structure-binding relationship studies revealed a promiscuous substrate (inhibitor) binding site inside the Pgp transmembrane part. It has great flexibility and also has larger than usual numbers of specificity residues. Instead, it can be described as large and deep pocket surrounded by clusters of hydrophobic residues. Therefore, it can accommodate less structurally related compounds. This binding site was proved to recognize, bind and efflux more than 300 diverse organic compounds belonging to chemotherapeutic and non-chemotherapeutic classes of drugs [12]. The Pgp efflux pump inhibitors may act through two different mechanisms: substrate site blockage and ATPase inhibition. In recent years, Pgp allosteric inhibitors (ATPase inhibitors) seemed more promising as several candidates (Tariquidar, Biricodar, Elacridar and Zosuquidar) are advancing in clinical trials (Fig 1) [13]. There are some skepticism if any of these compounds will eventually succeed to be the first-in-class MDR modulator to win approval as adjuvant in cancer chemotherapy due to intolerable side effects, unpredictable pharmacokinetics and drug-drug interactions [14]. Meanwhile, the direct pump inhibitors that bind to substrate site are logically a primary molecular mechanism for development of Pgp inhibitors. However, the studied compounds of this class, such as dexverapamil, caused problems during clinical trials [15]. Fortunately, many kinase inhibitors, such as vemurafenib, that were developed primarily as anticancer agents were found to directly block the ABCB1 (Pgp) as a secondary activity mechanism (Fig 1) [16].

There are many natural products that were characterized as MDR modulators through inhibition of ABCB1 [17, 18]. Curcumin is a versatile natural molecule that is possessing multitude of confirmed therapeutic activities but it is very safe and can be tolerated in high doses up to 10g/day [19, 20]. Regardless intensive research that confirmed the medicinal benefits of curcumin, the yellow condiment continues to intrigue more researchers to explore its activities on recently validated therapeutic targets [21–23]. Curcumin ameliorates response of cancer cells towards several cytotoxic drugs. Further investigations confirmed down-regulation of MDR-1b gene and hence, Pgp expression by curcumin via interruption of PI3K/Akt/NFκB pathway [24]. Due to high safety margin but low efficacy profile of curcumin, its analogues coming from natural sources (curcuminoinds) or from synthetic sources, were investigated for Pgp inhibitory activities. There are evidences that curcumin and its related compounds may act also through competitive or allosteric (ATPase) inhibition of Pgp in resistant cancer cell lines [25, 26].

The field of discovery of novel MDR modulators is anywhere but near closure [27]. In as much as MDR poses such disastrous failures in chemotherapy and hence, mortality, efforts must continue to discover improved MDR suppressors. Not separable, the impact of new molecular leads on multi-drug resistance against cytotoxic drugs should be on the center of the early in vitro studies. In this research, we are utilizing the structural architecture of curcumin to design compounds with confirmed Pgp inhibition activities and increased sensitivity of resistant cancer cells towards paclitaxel (PTX).

## Results

### Design of Pgp Inhibitors

The design of our compounds was based on flexibility of the SAR of curcumin derivatives as well as promiscuity of Pgp inhibition targets [25, 28, 29].

**Fig 2:**
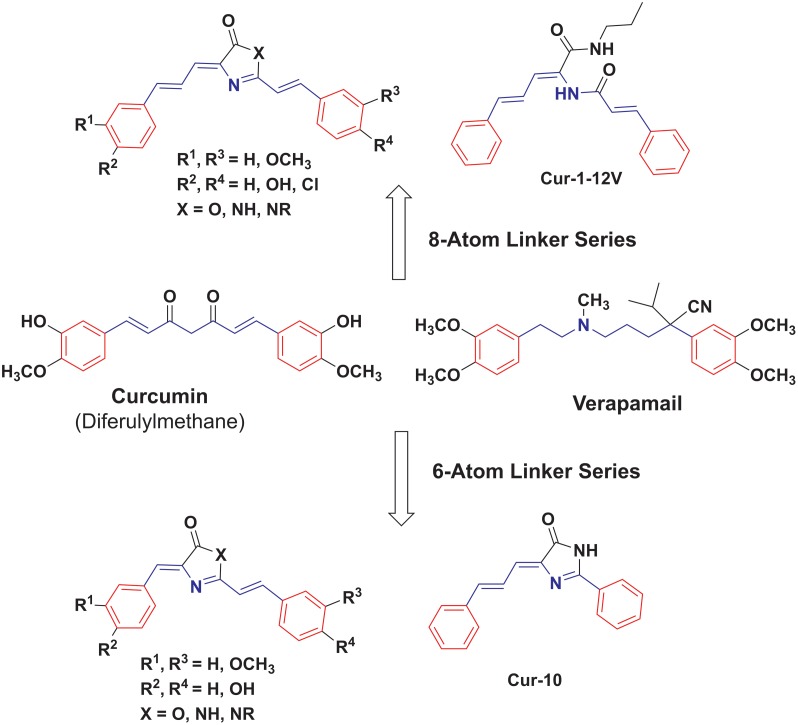
Design of Pgp inhibitors by re-scaffolding of the linker region of curcumin and verapamil.

Curcumin molecule (diferulylmethane) is composed of two distinct parts: A) Two terminal aromatic rings and B) Seven-atom linker with β-diketone in the center (Fig 2). The same main features are shared between three Pgp inhibitors: curcumin, tetrahydrocurcumin and verapamil (one of the earliest studied Pgp direct inhibitor). However, we acknowledge the differences in hybridization pattern that should affect the molecular geometry. Many of the reported analogues of curcumin are either arylvinylketones or bis-(arylvinyl)ketones. The curcumin analogues that did not adhere to the 7-atom linker rule kept good to excellent Pgp inhibition activities [30].

### Chemical Synthesis

**Fig 3:**
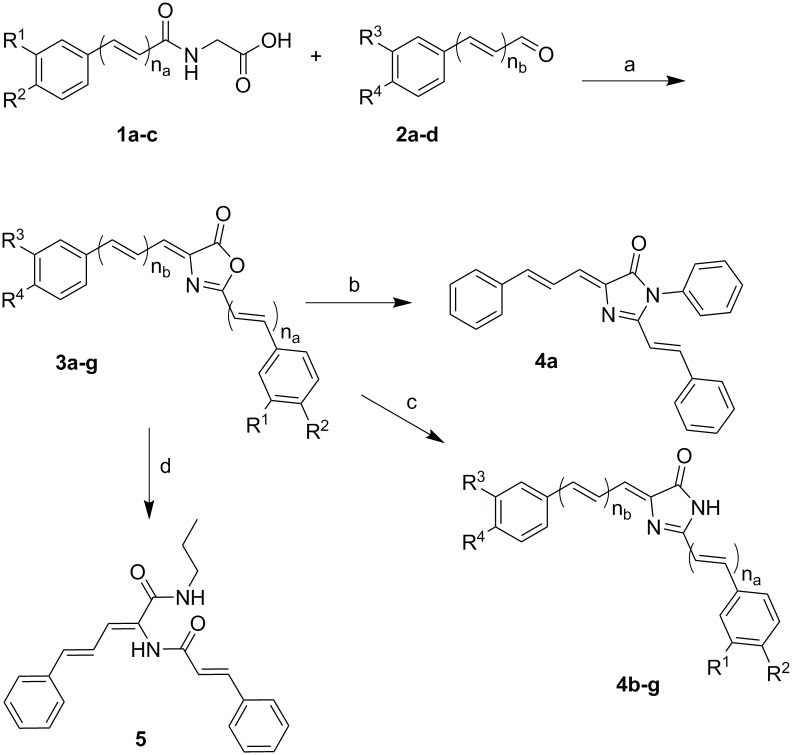
Chemical Synthesis Scheme. Reagents and Conditions: (a) Acetic anhydride, sodium acetate, heat. (b) Aniline, acetic acid, sodium acetate, 110°C, 24 hr. (c) Ammonium acetate, pyridine, 110°C, 18 hr. (d) *n*-PrNH_2_, EtOH, r.t., 2h.

The synthesis of the oxazolones **3a-g** followed standard Erlenmeyer chemistry for synthesis (Fig 3)[31]. The acylglycines **1a-c** were prepared in two steps by reaction of carboxylic acids with ethylglycinate followed by saponification. The acylglycines were condensed with aldehydes **2a-d** to furnish the oxa-analogues (4-oxazolones) **3a-g**. Some oxazolones were reacted with ammonium acetate in pyridine under anhydrous conditions to give the curcumin aza-analogues **4b-g**.

The *N*-phenylimidazolone **4a** was prepared using the oxazolone **3a** with aniline but the reaction medium contained acetic acid and sodium acetate [32]. The non-heterocyclic compound **5** was prepared accidentally as mentioned before. However, it was also systematically prepared by reaction of the oxazolone **3a** with excess *n*-propylamine in ethanol at room temperature. All new compounds (Table 1) were confirmed using NMR, IR and the molecular formulae were established using HRMS. The purities were confirmed using LC/MS.

**Table 1:** Structure and codes of the synthesized oxazolones and imidazolones.

Cpd No.	Code	R^1^	R^2^	n_a_	R^3^	R^4^	n_b_
**3a**	**Curox-1**	H	H	1	H	H	1
**3b**	**Curox-3**	H	H	1	OMe	OH	1
**3c**	**CinVox**	H	H	1	OMe	OH	0
**3d**	**FerVox**	OMe	OH	1	OMe	OH	0
**3e**	**-**	H	H	1	H	H	0
**3f**	**-**	H	H	0	H	H	1
**3g**	**-**	H	Cl	1	OMe	OH	1
**4a**	**Cur1-01**	H	H	1	H	H	1
**4b**	**Cur-03**	H	H	1	OMe	OH	1
**4c**	**Cur-07**	OMe	OH	1	OMe	OH	0
**4d**	**Cur-09**	H	H	1	H	H	0
**4e**	**Cur-10**	H	H	0	H	H	1
**4f**	**Cur-1**	H	H	1	H	H	1
**4g**	**Cur-8**	H	Cl	1	OMe	OH	0
**5**	**Cur1-12V**	H	H	1	H	H	1

### Biological Screening

#### Effect on purified recombinant P-glycoprotein (Pgp)

**Fig 4:**
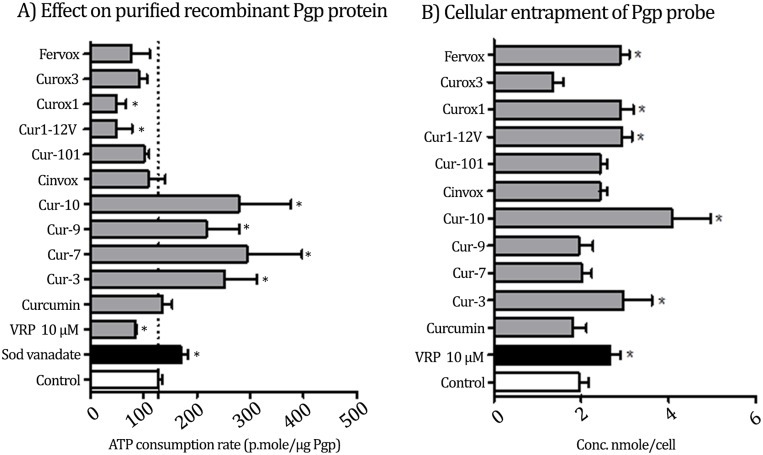
(A) The effect of test compounds on the activity of P-glycoprotein efflux pump cell free isolated recombinant P-gp protein and (B) within LS-174T cells. The influence of test compounds on the cellular pharmacokinetics via inhibiting the activity of Pgp pump. Verapamil (VRP) is a standard direct Pgp blocker. Sod Vanadate is a standard ATPase inhibitor. Curcumin is the prototype compound in the design. The asterisk (*) denotes significant difference from control.

First, the sub-molecular interaction between test compounds and Pgp subunits was undertaken using human recombinant Pgp membrane bound protein linked to ATPase enzyme subunits. It is known that Pgp inhibitors such as verapamil (VRP) are supposed to increase ATPase activity leading to more ATP consumption (33.1% less remaining ATP concentration compared to basal level). On the other hand, direct ATPase inhibitors such as sodium vanadate would decrease ATP consumption (33.9% more remaining ATP concentration compared to basal condition). Curox-1 (**3a**), and Cur1-12V (**5**) showed pure ATPase stimulatory effects with 61.4% and 62.2% less remaining ATP concentration, respectively. On the other hand, Cur-3, 7, 9 and 10 showed pure ATPase inhibition up because they significantly left more remaining ATP concentration compared to that left by basal ATPase activity (Fig 4a). Other curcumin analogues as well as curcumin itself did not induce any significant change for ATP consumption rate. This might be attributed to lack of interaction with either subunit of Pgp molecules or attributed to dual interaction with both subunits.

Multidrug resistance in particular tumor types, such as solid tumors within gastrointestinal tract, is highly attributed to impaired cellular pharmacokinetics and intracellular drug retention issues [33]. Therefore, our designed compounds to enhance the cellular entrapment of P-glycoprotein substrates were tested within LS-174T colorectal cancer cells. Curox-1 (**3a**), Fervox (**3c**), Cur-3 (**4b**), Cur-10 (**4e**) and Cur1-12V (**5**) significantly increased cellular retention of DOX (Pgp probe) and increased its intracellular concentration from 1.9±0.2 nmole/cell to 2.9±0.13 nmole/cell, 2.9±0.2 nmole/cell, 3.0±0.45 nmole/cell, 4.1±0.6 nmole/cell and 2.9±0.16 nmole/cell, respectively (Fig 4b). On the other hand, Curox-3, Cinvox, Cur1-01, Cur-7, Cur-9 and curcumin (reference compound) did not induce any significant increase for the intracellular DOX concentration. Compounds **4f** and **4g** were excluded from this test due to poor solubility at the assay conditions.

#### Cytotoxicity assessment of test compounds

Sulforhodamine B (SRB) assay was used to assess the cytotoxicity of newly synthesized compounds against Pgp high-expressing colorectal cancer cell line (LS-174T) over a concentration range 0.01–100 μM. LS-174T is known with its high resistance (high R-fraction) and consequently high probability of tumor recurrence [34]. This parameter is complimentary to the primary goal of inhibition of Pgp, accumulation of Pgp probe and modulation of resistance. Not all test compounds showed significant cytotoxicity against LS-174T cells. Nonetheless, some compounds exhibited considerably low IC_50_ values (less than 10 μM) accompanied with very low R-values (less than 5%). For instance, Curox-1 (**3a**), Curox-3 (**3b**), Fervox (**3c**), Cinvox (**3d**), Cur-10 (**4e**) and Cur1-12V (**5**) did not show good cytotoxicity since their IC_50_ values were higher than 100 μM. On the other hand, Cur-1, Cur-3 (**4b**), Cur-7 (**4c**), Cur-8 and Cur1-01 (**4a**) killed LS-174T cells with IC_50_ values of 7.6, 8.9, 35.9, 27.2 and 70.2 μM, respectively (Table 2). Interestingly, less than 5% of LS-174T cells were resistant to Cur-1, Cur-3, Cur-7 and Cur-8.

**Table 2:** Cytotoxic Activity of synthesized compounds against LS174T CRC cell lines.

Cpd No	Cpd Code	IC_50_ (μM)	(±)SE	R-fraction (%)
**3a**	Curox-1	>100	N/A	N/A
**3b**	Curox-3	>100	N/A	N/A
**3c**	Fervox	>100	N/A	N/A
**4a**	Cur1-01	70.2	2.7	N/A
**4b**	Cur-3	8.9	0.4	1.5
**4c**	Cur-7	35.9	2.6	0.0
**4d**	Cur-9	N/T	N/A	N/A
**4e**	Cur-10	>100	N/A	N/A
**4f**	Cur-1	7.6	1.3	0.8
**4g**	Cur-8	27.2	6.1	0.0
**5**	Cur1-12V	>100	N/A	N/A

#### Chemomodulatory effect of Cur-3 and Cur1-12V to paclitaxel (PTX) within colorectal cancer cells

**Fig 5:**
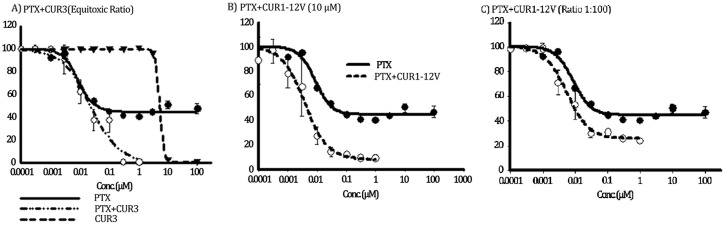
The effect of serial equitoxic concentrations (ratio 1:100) of Cur3 (A), and 10 μM (B), and serial concentration of Cur1-12V (ratio 1:100) (C) on the cytotoxicity of PTX in LS-174T cells. Cells were exposed to serial dilution of PTX (●), Chemosensetizer (▼) or their combination (○) for 72 h. Cell viability was determined using SRB assay.

Attributed to their clear Pgp interaction properties as well as their considerable effect on Pgp substrate cellular accumulation, Cur-3 (ATPase inhibitor, cytotoxic) and Cur1-12V (Direct Pgp inhibitor, no cytotoxic activity) would be good candidates to improve the activity of Pgp substrate drugs (such as paclitaxel) within Pgp expressing tumor cell types (such as LS-174T colorectal cancer cells). Regardless the significant cytotoxic activity of Cur-3, it is a thousand fold weaker cytotoxic than PTX (7.9±0.5 nM). In combination with PTX, Cur-3 (**4b**) did not increase the potency of PTX against LS-174T (Fig 5a). This can be observed from a slight increase of IC_50_ of PTX to 23.8±8.1 nM. It can be even declared that Cur-3 antagonized the potency of PTX against LS-174T with combination index value equals 3.27. However, Cur-3 abolished the resistance of LS-174T to PTX from R-value of 45.7±3.6% to 2.5±1.1%. Conversely, the Pgp direct competitive inhibitor Cur1-12V (10 μM) significantly increased PTX potency against LS-174T cells; IC_50_ values of PTX were 7.9±0.5 nM and 4.3±1.1 nM alone and in combination with Cur1-12V, respectively (Fig 5b). In addition, Cur1-12V significantly decreased the resistance of LS-174T to PTX from R-value of 45.7±3.6% to 16.2±6.6% for PTX alone and in combination with Cur1-12V, respectively (Fig 5). A similar result was observed in a combination of Cur1-12V with PTX at the same ratio used for Cur-3 (Table 3).

**Table 3:** Chemomedulatory effects of test compounds on the cytotoxicity parameters of PTX against LS-174T cell line.

Compound	Cytotoxic parameter			
	LS-174T cells		HCT-116 cells	
	IC_50_	R-fraction (%)	IC_50_	R-fraction (%)
**PTX**	7.9±0.5 nM	45.7±3.6%	102.7±12.7 nM	43.9±1.7%
**Cur-3**	8.9±0.4 μM	0.92±0.5%	12.23±1.6 μM	4.1±1.1%
**Cur1-12V**	>100 μM	N/A	>100 μM	N/A
**PTX+Cur-3 (1:100)**	23.8±8.1 nM	2.5±1.1%	63.6±4.2 nM	2.7±1.1%
**PTX+Cur1-12V (10 μM)**	4.3±1.1 nM	16.2±6.6%	12.6±2.9 nM	40.4±1.6%
**PTX+Cur1-12V (1:100)**	4.9±0.9 nM	28.1±2.1%	17.5±3.1 nM	38.6±1.9%

**Fig 6:**
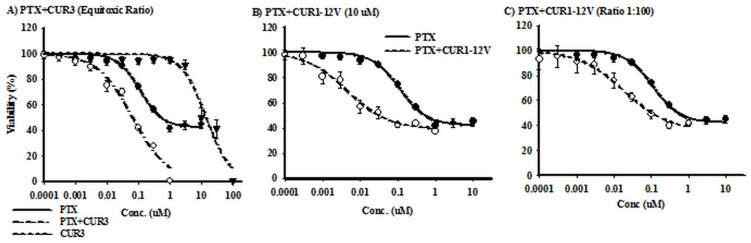
The effect of serial equitoxic concentrations (ratio 1:100) of Cur3 (A), and 10 μM (B), and serial concentration of Cur1-12V (ratio 1:100) (C) on the cytotoxicity of PTX in HCT-116 cells. Cells were exposed to serial dilution of PTX (●), Chemosensetizer (▼) or their combination (○) for 72 h. Cell viability was determined using SRB assay.

Similar to LS-174T cell line, Cur-3 exerted humble cytotoxic effect against HCT-116 cells (12.23±1.6 μM) compared to PTX (102.7±12.7 nM). However, Cur-3 killed HCT-116 with very low resistance remaining fraction (4.1±1.1%). On the other hand, Cur1-12V did not manage to kill HCT-116 up to 100 μM for 72h. Ultimately, PTX faced resistance fraction up to 43.9±1.7% within HCT-116 cells (Fig 6). Interestingly, equitoxic combination of Cur-3 enhanced the potency of PTX against HCT-116 (63.6±4.2 nM) as well as decreased the resistance fraction to 2.7±1.1% (Fig 6a). Additionally, Cur1-12V (10 μM) increased the potency of PTX against HCT-116 cells (12.6±2.9 nM); while it did not significantly influence their resistance fraction (40.4±1.6%) (Fig 6b). Similar results was observed when combining Cur1-12V with PTX using 1:100 ratio (Fig 6c).

## Discussion

Our objective of this research was to introduce molecular frameworks supported by a simple, yet efficient, protocol for *in vitro* screening of MDR modulators. Our design was inspired by curcumin because it is established chemosensitizer as supported by several researches. The re-scaffolding process of the central part furnished compounds with good activity in chemosensitazation of resistant CRC cancer cells against PTX. The screening protocol was designed as follows: First to identify Pgp inhibitors by measuring the ATP consumption rate. This method is useful not only in quantifying the Pgp inhibition, but also in identifying the mechanism of action. This assay is sub-cellular, therefore, the protein expression inhibitors are excluded from its scope. The test could differentiate between compounds that bind directly to the efflux substrate site and those acting on ATPase subunit leading to deprivation of the efflux pump from necessary energy source. Complimentary and parallel to ATP consumption test, we conducted cellular entrapment test that measures the accumulation of a Pgp probe (substrate) inside cells that are overexpressing Pgp. The short incubation time (2 h) also eliminated the possibility of protein expression mechanism. The ATPase activity test gives an indication of binding to Pgp and its inhibition; but the entrapment test answers the question whether this binding is translated into accumulation of a drug inside the resistant cells. Combining the results of the two tests, from our perspective, is a short way to decide which compounds should be the focus of next investigations (Combination with PTX). We determined that a successful candidate should lead to accumulation of Pgp substrate (probe) inside Pgp expressing cells significantly. In addition such a compound should show a confirmed Pgp inhibition through a defined mechanism as interpreted from the ATP consumption test. Our observation was that five (out of 10) test compounds increased cellular permeability of DOX. However, only Curox-1, Cur-3, Cur-10 and Cur1-12V met the criteria because they either significantly increased ATP consumption (Curox-1 and Cur1-12V) or decreased ATP consumption (Cur-3 and Cur-10). Curox-1 and Fervox increased cellular retention of DOX. However, they were excluded because their effect on the purified Pgp was not clear, as there was no significant change in ATP consumption rate compared to control. Our interpretations is that these two compounds either act as dual inhibitors (inhibit both substrate site and the ATPase simultaneously), or they inhibit MDR transporter(s) other than Pgp.

The structure-activity relationship (SAR) for activities on the purified Pgp protein is clear. The subclass oxazolones (Curox-1, Curox-3, Cinvox and Fervox) generally preferred the Pgp substrate site over the ATPase subunit. All of these compounds increased ATP consumption rate either significantly (Curox-1) or slightly (others). All the compounds that have unsubstituted nitrogen (NH) at position 1 of the imidazolone ring (Cur-3, 7, 9 and 10) showed obvious affinity towards the ATPAse subunit indicating suitability of the presence of hydrogen bond donor at this position for binding. Meanwhile, the *N*-phenyl analogue (Cur1-01) was not significant inhibitor of the Pgp and did not enhance the cellular entrapment of DOX either, indicating bulk intolerance at this position of the heterocyclic ring. The open non-heterocyclic analogue Cur-12V was unique structure in the tested set of compounds. It showed also direct inhibition of the Pgp (similar to oxazolone analogues e.g. Curox-1) as well as significant increase in the retention of DOX. This interesting compound is planned for further studies because it has obvious drug-like structure. Other than clear SAR among different core structures, the length of the linker (6-atom *vs*. 8-atom series) had smaller impact on activities within the same chemical species.

The cytotoxicity of the synthesized compounds is of secondary importance after their Pgp inhibitory and chemomodulatory influence. However, the cytotoxic activities of test compounds alone should be in consideration upon running the combination tests with PTX. It was interesting to find that SAR pattern of the cytotoxic activities is similar to that of Pgp activities. The ATPase-inhibitory NH imidazolone series Cur-1, 3, 7 and 9 exhibited good to moderate antiproliferative effects against LS-174T CRC cells. Furthermore, some of them (Cur-1 and Cur-3) showed low micromolar IC_50_ at 7.6 and 8.9 μM respectively. Not surprisingly, the R-value that measures the level of resistance was very low in all cases. This is expected because LS-174T cells are over expressing Pgp and possess inherent resistance to cytotoxic agents. Our finding is that the cytotoxic compounds (Cur-3 and 7) might be considered as auto-chemosensitizers, causing accumulation of their own molecular populations inside cancer cells. On the other hand, the oxazolones as well as the open non-heterocyclic analogue Cur1-12V did not show any significant anticancer activities under 100 μM. Thus, it was interesting to compare the effect of both compounds (Cur-3 and Cur1-12V), as representatives of two distinct classes, on the resistance of LS-174T CRC cell lines. Cur-3 (**4b**) embodies the ATPase inhibitor subclass, causes accumulation of Pgp probe and has considerable cytotoxicity at low micromolar level. Cur12-V embodies direct Pgp inhibitor subclass, also causes considerable retention of Pgp probe but it is not cytotoxic. For this assay, we ignored all compounds that showed lower potency in cellular entrapment assay (Curox-3, Cinvox, Cur1-01, Cur-7 and Cur-9), caused color interference with assays (Curox-1) or suffered from poor solubility (Cur-1, Cur-8 and Cur-10).[35, 36]

Paclitaxel (PTX) is a commonly used chemotherapeutic agent as first line treatment of many solid tumors.[37] However, its efficacy in gastrointestinal tumors is hampered by MDR type of resistance.[38–40] For instance, our assay showed that PTX is quite potent against S-174T CRC cancer cell lines with IC_50_ at nanomolar level (7.9±0.5 nM) but its resistance fraction is very high (R-value is 45.7±3.6%). Unlike other reports, our research was focused on the effect of the chemosensitizers Cur-3 and Cur1-12V on the R-value when given in combination with PTX.[41–46]. This is because the decrease in the IC_50_ does not necessarily mean that the resistance is subsequently suppressed. However, the change in IC_50_ should not be ignored to guard against antagonistic activities and negative drug-drug interactions. Combinations of PTX with test compounds resulted in diminishing the resistance fraction from 45.7% to 2.5% and 16.2% for Cur-3 and Cur1-12V, respectively. Since PTX is proven to be a substrate to Pgp efflux pump, the resistance change is likely due to the impact of our compounds on the Pgp (both proven to inhibit the Pgp). To confirm results, we repeated the same combination test protocol against another resistant CRC cell line, the HCT-116. Similar results were obtained except that CUR1-12V decreased the IC_50_ but did not significantly affect the resistant fraction. Inhibition of other MDR proteins such as MRP1 and BCRB should be included in future investigations as they were not in the scope of this particular research. Nonetheless, our compounds clearly caused modulation of the MDR leading to sensitization of the tumor cells towards PTX.

## Conclusion

Our design, based on curcumin as lead and safe natural product, furnished serious new molecular frameworks to modulate multidrug resistance as potential adjuvant treatment of tumors overexpressing Pgp. The initial investigation, though simple, introduced a model of how to quickly and effectively quantify the chemosensitizing properties of new compounds based on changes in resistance (primarily) and potency (conjunctively).

Indeed it is too early to state that we finished investigation of our two lead compounds as further studies should be conducted on utility on non-Pgp expressing cells as well as different resistant cell lines. Our scope was to build a framework (compounds and efficient assay) as a start. The two lead compounds, either the cytotoxic Cur-3 or the non-cytotoxic Cur1-12V, should be subjected to further studies for optimization and development as MDR modulators. Just to mention some, future investigations should include effect on other types of cancer that are over expressing as well as those not expressing (Pgp-knockout) cells. Effect of our compounds on Pgp protein expression (curcumin-like effect), and inhibition assay against other MDR protein such as MRP-1 and BCRP are important to clarify the scope and the exact mechanism of the newly discovered lead compounds.

## Experimental

Melting points (m.p.) were determined in open capillary tubes using Electrothermal apparatus (Stuart, UK) and are uncorrected. IR spectra were recorded using the potassium bromide method on Perkin-Elmer 1650 spectrophotometer and expressed in wave number (υmax) cm−1.). In this report, we only listed the important IR stretching bands, including NH, OH, CH, C = O, C = N and/or C = C. 1H-NMR and 13C-NMR spectra were measured in deuterated chloroform (CDCl3) or deuterated dimethyl sulphoxide (DMSO-d6), on Avance III 850 MHz, Avance III 600 MHz or AV-400 MHz spectrometers (Bruker, Billerica, Massachusetts, USA), at the Faculty of Science Spectroscopy Center, King Abdulaziz University, Jeddah, Saudi Arabia. Chemical shifts were expressed as ppm against TMS as internal reference. LC/MS analyses were performed on an Agilent 6320 Ion Trap HPLC–ESI-MS/DAD (Santa Clara, CA, USA) with the following settings: The analytes were separated using an Macherey-Nagel Nucleodur-C18 column (150 mm length × 4.6 mm i.d., 5 μm) (Macherey-Nagel GMBH & Co. KG, Duren, Germany). Mobile phase; isocratic elution using a mixture of isopropanol and 0.01M ammonium acetate in water (65: 35, v/v). The flow rate was 0.4 mL/min.; total run time = 20 min. Purities are reported according to percentage of Peak Areas at wavelength 244 nm. Reaction progress and compound purity were monitored by Thin Layer Chromatography (TLC) using silica gel matrix, L × W 5 cm × 20 cm, fluorescent indicator (Machery-Nagel Co., Germany). Column chromatography was performed on silica gel 60 (particle size 0.06 mm—0.20 mm). High-resolution mass spectrometry (HRMS) was performed in the Faculty of Science, King Abdulaziz University on Impact II^™^ Q-TOF spectrometer (Bruker, Germany). All fine chemicals and solvents were purchased from the authorized local distributors of Sigma Aldrich (USA), Acros (Belgium), Alfa-Aeser (England) and Panreac (Spain). Some reagents were purchased and imported from Molport (England). Sulforhodamine-B was purchased from Sigma-Aldrich (St. Louis, MO, USA). Tricloroacetic acid (TCA) and other materials used in biological screening were of the highest available commercial grade. Vacuum evaporation were performed on Büchi Rotavapor R-215. Solvent and chemicals waste were collected and disposed according to KAU Laboratory Safety and Environmental Protection Guidelines.

### Chemical Synthesis

Acylglycines **1a-c** (all are reported compounds) are prepared according to known procedure of amide formation by reaction of (*E*)-acid chlorides and ethyl glycinate hydrochloride followed by saponification of the product with NaOH at 40°C. Compounds **3e**,[47] **3f**,[48] and **4d**[49] were prepared according to general procedures mentioned below in the synthesis of other new compounds of the this research and their spectral data were found identical to that of literatures. The intermediate **3g** was prepared and used directly without further characterization for the next step.

#### General procedure for preparation of oxazolones 3a-3g

A mixture of the (*E*)-arylglycine (0.01 mol), and the (*E*)-arylcarbaldehyde (2a) (0.011 mol), anhydrous sodium acetate (0.05 g) and acetic anhydride (5ml) was warmed on a boiling water bath with occasional stirring for 30 h, cooled and left at 4°C for 18 h. The produced yellow solid mass was filtered under vacuum until complete dryness. The solid was washed with cold ethanol-water mixture, the solvent was removed and the solid was dried by vacuum filtration for sufficient time. The solid was washed twice with petroleum ether and the crude product was crystalized from ethanol.

#### (Z)-4-[(E)-3-phenylallylidene]-2-[(E)-styryl)oxazol-5(4H)-one (3a)

This compound was prepared in 83% yield as a yellow solid, m.p. 150–151°C.[50] ^1^H NMR (850 MHz, DMSO-*d*_6_) δ 7.78–7.86 (m, 1H), 7.71 (d, *J* = 16.09 Hz, 1H), 7.67 (d, *J* = 7.27 Hz, 2H), 7.60–7.65 (m, 1H), 7.55 (dd, *J* = 11.42, 15.57 Hz, 1H), 7.44–7.50 (m, 5H), 7.35–7.44 (m, 2H), 7.21 (d, *J* = 11.42 Hz, 1H), 7.08 (d, *J* = 16.61 Hz, 1H); IR (FT-IR, cm^−1^): 3053.13, 1775.82, 1639.51, 1591.55, 1563.78, 1452.71, 1316.4; LC-MS (ESI), RT = 13.4 min, *m/z* 301.9 [M + H]^+^.

#### (Z)-2-[(E)-4-hydroxy-3-methoxystyryl]-4-[(E)-3-phenylallylidene]oxazol-5(4H)-one (3b)

This compound was prepared in 83% yield as a yellow-brown solid in 70% yield; m.p. 149–151°C. ^1^H NMR (850 MHz, DMSO-*d*_6_) δ 7.69 (d, *J* = 16.09 Hz, 1H), 7.60–7.67 (m, 3H), 7.44–7.50 (m, 3H), 7.35–7.44 (m, 3H), 7.14–7.21 (m, 2H), 3.80–3.94 (m, 4H); IR (FT-IR, cm^−1^): 3060.7, 3005.17, 1758.15, 1644.56, 1614.27, 1503.2, 1417.37, 1273.48, 1200.28; LC-MS (ESI), RT = 7.7 min, *m/z* 347.9 [M + H]^+^.

#### 4-[(Z)-4-hydroxy-3-methoxybenzylidene)-2-[(E)-styryl]oxazol-5(4H)-one (3c)

This compound was prepared in 83% yield as a yellow solid powder was obtained in 61% yield, m.p. >250°C (dec). ^1^H NMR (850 MHz, CDCl_3_) δ 8.00–8.05 (m, 1H), 7.73 (d, *J* = 16.09 Hz, 1H), 7.62–7.66 (m, 3H), 7.42–7.50 (m, 3H), 7.18 (s, 1H), 7.15 (d, *J* = 7.78 Hz, 1H), 6.84 (d, *J* = 16.09 Hz, 1H), 3.95–4.03 (m, 3H); IR (FT-IR, cm^-1^): 1763.2, 1642.03, 1599.12, 1513.29, 1422.42, 1311.35, 1265.91, 1200.28; LC-MS (ESI), RT = 8.5 min, *m/z* 321.9 [M + H]^+^; ^13^C NMR (214 MHz, CDCl_3_) δ 168.7, 167.3, 163.4, 151.4, 144.1, 142.2, 134.6, 133.5, 132.5, 130.9, 130.4, 129.1, 128.2, 126.0, 123.2, 115.4, 113.4, 56.0, 20.7.

#### 4-[(Z)-4-hydroxy-3-methoxybenzylidene]-2-[(E)-4-hydroxy-3-methoxystyryl]oxazol-5(4H)-one (3d)

This compound was prepared in 83% yield as a yellow solid in 77% yield, m.p. 174–176°C. ^1^H NMR (850 MHz, CDCl_3_) δ 7.98 (s, 1H), 7.64–7.71 (m, 2H), 7.20–7.26 (m, 1H), 7.06–7.20 (m, 3H), 6.77 (d, *J* = 16.09 Hz, 1H), 3.95–4.09 (m, 3H), 3.87–3.95 (m, 3H), 3.85 (s, 1H); IR (FT-IR, cm^-1^): 2942.06, 2848.66, 1785.92, 1758.15, 1649.61, 1594.07, 1513.29, 1417.37, 1369.41, 1270.96; LC-MS (ESI), RT = 5.0 min, *m/z* 368.0 [M + H]^+^; ^13^C NMR (214 MHz, CDCl_3_) δ 168.8, 168.7, 167.2, 163.3, 151.6, 151.4, 143.3, 142.2, 141.9, 133.5, 133.5, 132.5, 130.6, 125.9, 124.8, 123.5, 123.4, 123.4, 123.2, 121.6, 121.5, 115.4, 113.6, 111.4, 111.3, 56.1, 56.1, 56.0, 56.0, 20.7, 20.7.

#### Preparation of (Z)-3-phenyl-5-[(E)-3-phenylallylidene]-2-(E)-styryl-3,5-dihydro-4H-imidazol-4-one (4a)

The oxazolone **3a** (1.5 g, 0.005 mol) was dissolved in 15 mL acetic acid containing 0.5 g sodium acetate and heated. Aniline (0.512 g, 0.5 mL, 0.0055 mol) added to the heated mixture and temperature was adjusted to 100°C for 8 hrs. Most of solvent was removed by vacuum evaporation and the residue was poured into ice water containing 10% of HCl. The precipitated solid was collected by vacuum filtration, washed with water, sodium bicarbonate then water. The dried crude solid was purified by column chromatography (gradient: hexane to hexane-dichloromethane to dichloromethane) to afford 0.43 g (23%) of pure product **4a** as page yellow solid, m.p. 180–185°C. ^1^H NMR (850 MHz, DMSO-d_6_) δ 7.93 (d, *J* = 15.57 Hz, 1H), 7.69 (d, *J* = 7.27 Hz, 2H), 7.57–7.63 (m, 3H), 7.51–7.57 (m, 2H), 7.36–7.50 (m, 10H), 7.11 (d, *J* = 11.42 Hz, 1H), 6.59–6.65 (m, 1H); IR (FT-IR, cm^−1^): 3058.18, 3027.89, 2995.07, 1705.14, 1621.84, 1576. 40, 1495.62, 1379.50, 1331.54, 1210.37; LC-MS (ESI), RT = 13.9 min, *m/z* 377.0 [M + H]^+^.

#### Parallel synthesis of compounds 4b-4g

Anhydrous ammonium acetate (0.46 g, 0.005 mol) was placed 25 mL-tube of Thermal Integrity Reaction Station, added the appropriate azlactone (0.003 mol) and pyridine (10 ml) under inert air. The device was adjusted for 18 h run at 100°C for 18 and stirring. The solvent was removed by evaporation under vacuum. The residue was subjected to flash column chromatography (silica gel, mixtures of petroleum ether/dichloromethane, 1:4, v/v) to afford the desired pure compounds **(4b-g)**.

#### (Z)-5-[(E)-3-(4-hydroxy-3-methoxyphenyl)allylidene]-2-(E)-styryl-3,5-dihydro-4H-imidazol-4-one (4b)

The product was dark red solid and it was obtained in 55% yield, m.p. 97–100°C. ^1^H NMR (600 MHz, DMSO-*d*_6_) δ 7.52–7.72 (m, 3H), 7.32–7.51 (m, 4H), 7.14–7.26 (m, 2H), 6.94–7.14 (m, 2H), 6.76–6.94 (m, 2H), 3.76–3.96 (m, 3H); IR (FT-IR, cm^−1^): 3060.71, 2934.49, 2838.57, 1768.25, 1712.71, 1642.03, 1578.92, 1465.33, 1445.14, 1432.51, 1116.97; High Resolution MS (EI^+^, *m/z*) (M+H)^+^ Calcd. for C_21_H_19_N_2_O_3_, 447.1351, found 447.1382. ^13^C NMR (214 MHz, DMSO-*d*_6_) δ 170.7, 158.3, 149.1, 148.5, 144.5, 143.8, 143.0, 140.7, 139.9, 139.8, 135.5, 135.2, 130.8, 130.5, 130.0, 129.6, 129.6, 129.5, 129.4, 128.7, 128.5, 128.3, 128.2, 128.1, 128.0, 122.6, 121.2, 119.6, 117.5, 116.3, 110.7, 56.0, 49.0.

#### 5-[(Z)-4-hydroxy-3-methoxybenzylidene)-2-[(E)-4-hydroxy-3-methoxystyryl]-3,5-dihydro-4H-imidazol-4-one (4c)

The product was brick red and it was obtained in 53% yield, m.p 113–116°C. ^1^H NMR (850 MHz, DMSO-d_6_) δ 7.87–8.00 (m, 1H), 7.72–7.83 (m, 1H), 7.58 (d, *J* = 16.09 Hz, 1H), 7.45–7.52 (m, 1H), 7.20–7.27 (m, 1H), 7.14 (s, 1H), 6.87–6.98 (m, 2H), 6.78–6.87 (m, 1H), 3.80–3.92 (m, 6H); IR (FT-IR, cm^−1^): 2936.76, 2844.68, 1594.22, 1509.4, 1429.43, 1269.49, 1208.91; LC-MS (ESI), RT = 3.0 min, *m/z* 367.9 [M + H]^+^. High Resolution MS (EI^+^, *m/z*) (M+H)^+^ Calcd. for C_20_H_19_N_2_O_5_, 367.1288, found 367.1290.

#### 5-(Z)-benzylidene-2-(E)-styryl-3,5-dihydro-4H-imidazol-4-one (4d)

The product was yellowish-brown solid obtained in 42% yield. ^1^H NMR (600 MHz, DMSO-*d*_6_) δ 7.62 (d, *J* = 7.53 Hz, 2H), 7.52–7.58 (m, 1H), 7.51 (s, 1H), 7.35–7.48 (m, 5H), 7.26–7.35 (m, 1H), 7.16–7.24 (m, 1H), 7.02–7.12 (m, 1H), 6.87 (dd, *J* = 1.88, 15.81 Hz, 1H); High Resolution MS (EI^+^, *m/z*) molecular ion requires for C_18_H_14_N_2_O, 275.1140, found 275.1176.

#### (Z)-2-phenyl-5-[(E)-3-phenylallylidene]-3,5-dihydro-4H-imidazol-4-one (4e)

The product was yellowish crystals and it was obtained in 55% yield. ^1^H NMR (850 MHz, DMSO-*d*_6_) δ 8.30–8.43 (m, 3H), 8.19 (d, *J* = 7.79 Hz, 2H), 7.67 (t, *J* = 7.01 Hz, 1H), 7.62 (t, *J* = 7.27 Hz, 2H), 7.57 (d, *J* = 8.30 Hz, 2H), 7.00–7.10 (m, 3H); IR (FT-IR, cm^−1^): 3154.11, 3116.24, 3060.71, 1700.09, 1642.03, 1591.55, 1493.10, 1450.19, 1359.31, 1263.39; LC-MS (ESI), RT = 4.2 min, *m/z* 301.

#### (Z)-5-[(E)-3-phenylallylidene]-2-(E)-styryl-3,5-dihydro-4H-imidazol-4-one (4f)

The product was yellowish solid and it was obtained in 59% yield; m.p. 243–244°C. ^1^H NMR (850 MHz, DMSO-*d*_6_) δ 11.80 (s, 1H), 11.74 (s, 1H), 7.68 (d, *J* = 7.27 Hz, 1H), 7.64–7.67 (m, 1H), 7.61–7.64 (m, 1H), 7.54–7.61 (m, 2H), 7.40–7.50 (m, 4H), 7.35–7.39 (m, 1H), 7.27 (d, *J* = 15.57 Hz, 1H), 7.00 (d, *J* = 16.61 Hz, 1H), 6.87–6.94 (m, 1H); IR (FT-IR, cm^−1^): 3138.96, 3085.95, 3025.37, 1695.04, 1639.51, 1601.64, 1571.35, 1525.91, 1442.61, 1316.39, 1276.01, 1202.80; LC-MS (ESI), RT = 4.2 min, *m/z* 30.

#### 2-(E)-4-chlorostyryl-5-[(Z)-4-hydroxy-3-methoxybenzylidene]-3,5-dihydro-4H-imidazol-4-one (4g)

The product was yellowish brown solid and it was obtained in 78% yield. ^1^H NMR (600 MHz, DMSO-*d*_6_) δ ppm 3.85 (s, 3 H) 6.87 (d, *J* = 7.91 Hz, 1 H) 6.92 (s, 2 H) 7.04 (d, *J* = 16.56 Hz, 1 H) 7.52 (d, *J* = 8.28 Hz, 1 H) 7.60 (d, *J* = 16.56 Hz, 1 H) 7.64–7.79 (m, 2 H) 8.02 (s, 1 H); High Resolution MS (EI^+^, m/z) molecular ion requires for, C_19_H_15_ ClN_2_O_3_, 355.0805, found 355.0852.

#### (2E,4E)-2-cinnamamido-5-phenyl-N-propylpenta-2,4-dienamide (5)

The azlactone **3a** (0.01 mol) was placed in 20 mL ethanol and propyl amine (0.02 mol) and the mixture was left to stir at r.t. for 2 h. After completion of the reaction as monitored by TLC (2% MeOH in dichloromethane), the precipitated solid compound **5** was collected by filtration, washed with ethanol and crystallized from a mixture of dicloromethane and methanol. The yellowish crystalline solid was obtained in 61% yield; m.p. 205–207°C. ^1^H NMR (850 MHz, DMSO-d_6_) δ 9.68 (s, 1H), 8.02 (dd, *J* = 5.45 Hz, 1H), 7.64 (d, *J* = 7.27 Hz, 2H), 7.50–7.56 (m, 3H), 7.45–7.49 (m, 2H), 7.40–7.45 (m, 1H), 7.34–7.40 (m, 2H), 7.25–7.32 (m, 1H), 7.02 (dd, *J* = 11.42, 15.57 Hz, 1H), 6.83–6.94 (m, 2H), 6.72 (d, *J* = 11.42 Hz, 1H), 3.10 (q, *J* = 6.75 Hz, 2H), 1.47 (sxt, *J* = 7.27 Hz, 2H), 0.87 (t, *J* = 7.53 Hz, 3H); IR (FT-IR, cm^-1^): 3367.08, 2956.22, 2931.57, 2876.79, 2057.8, 1657.9, 1644.2, 1614.07, 1531.9, 1482.6, 1329.21, 1279.91, 1211.43; LC-MS (ESI), RT = 3.3 min, *m/z* 361.0 [M + H]^+^.

### Biological Screening

#### Cell culture

Human colorectal cancer cell line (LS-174T), was generously gifted from Professor Vladimir P. Torchilin (Northeastern University, Boston, MA, USA); Human colorectal cancer cell line (HCT-116 was obtained from VACCERA, Giza, Egypt. Both cell lines were maintained in RPMI-1640 media; supplemented with 100 μg/mL streptomycin, 100 units/mL penicillin and 10% heat-inactivated fetal bovine serum. Cells were propagated in a humidified incubator at 37°C with 5% (v/v) CO_2_ atmosphere.

#### Assessment of Pgp activity in-situ

To assess the effect of test compounds on the efflux pumping activity of Pgp in tumor cells, the Pgp substrate, doxorubicin (DOX), was used as a probe and verapamil (VRP) was used as standard Pgp blocker. Briefly, exponentially growing cells were plated in 6-well plates in plating density of 10^5^cells/well. Cells were exposed to DOX (10 μM), DOX (10 μM) and test compound (10 μM), or DOX (10 μM) and VRP (10 μM) for 2 h at 37°C and subsequently extracellular DOX containing media was washed trice with ice cold PBS. Intracellular DOX were extracted after cell lysis by incubation with SDS (2% w/v), saturated aqueous solution of ZnSO_4_ (100 μl), Acetonitrile (500 μl) and acetone (500 μl) for 30 min at 37°C. After centrifugation, supernatant was collected and DOX concentration was measured spectroflourometrically at λ_ex/em_ 482/550 nm [43, 51].

#### Assessment of Pgp ATPase activity

Assessment of Pgp-attached ATPase activity was performed using Pgp-Glo^™^ Assay Systems (Promega Corporation, Madison, Wisconsin, USA). Briefly, in 96-well plates, Pgp-Glo^™^ Assay Buffer was mixed with sodium vanadate, VRP, or test compound (10 μM). Recombinant human Pgp membrane fraction was incubated with the reaction mixture at 37°C for about 5 minutes, and then the reaction was initiated by adding 10μl of 25mM MgATP and incubating for 40 minutes at 37°C. An identical reaction mixture without drug treatment (NTC) was assayed in parallel. The reaction was stopped and the remaining un-consumed ATP was detected using luciferase firefly luminescent signal and ATP standard curve was plotted. The remaining concentrations of ATP were expressed as (p. mole/μg Pgp molecules). Sodium vanadate (Na_3_VO_4_) was used as selective inhibitor of Pgp related ATPase enzyme, and samples treated with Na_3_VO_4_ have greater luminescent signal than un-treated samples (NTC). In addition, VRP inhibits pgp molecules via covalent binding and competing with other substrates, hence, stimulating ATPase activity resulting in more consumption of ATP molecules compared to NTC.

#### Cytotoxicity assays

The cytotoxicity of the test compounds were tested against exponentially growing LS-174T cells by sulforhodamine B (SRB) assay as previously described [52]. Briefly, sub-confluent cells were collected using 0.25% Trypsin-EDTA and plated in 96-well plates at 1000–2000 cells/well. Cells were exposed to test compound for 72 h and subsequently fixed with TCA (10%) for 1 h at 4°C. After washing trice, cells were exposed to 0.4% SRB solution for 10 min in dark and subsequently washed with 1% glacial acetic acid. After drying overnight, Tris-HCl was used to dissolve the SRB-stained cells and color intensity was measured at 540 nm.

The dose response curve of each compound was analyzed using E_max_ model:

$$\%Cell\;viability=\left(100-R\right)\times \left(1-\frac{{\left[D\right]}^{m}}{K_{d}{}^{m}+{\left[D\right]}^{m}}\right)+R$$

Where [R] is the residual unaffected fraction (the resistance fraction), [D] is the drug concentration used, [K_d_] or IC_50_ is the drug concentration that produces a 50% reduction of the maximum inhibition rate and [m] is a Hill-type coefficient. Absolute IC_50_ is defined as the drug concentration required to reduce absorbance by 50% of that of the control (i.e., K_d_ = absolute IC_50_ when R = 0 and E_max_ = 100-R) [43].

#### Chemomodulatory effect of CUR3 and CUR1-12V to paclitaxel within colorectal cancer cells

The chemomodulatory effect of the non cytotoxic compound, CUR1-12V, to paclitaxel (PTX) within colorectal cancer cells was determined using the Pgp inhibitory concentration of CUR1-12V (10 μM) along with serial concentrations of PTX. Chemomodulatory effect of CUR3 to PTX within was determined using equitoxic combination analysis between PTX and CUR3 (exposure ratio 1:100) as previously described [53]. In addition, chemomodulatory effect of CUR1-12V to PTX at exposure ratio of 1:100 was assessed as well for comparison with CUR3. Briefly, exponentially growing cells were seeded in 96-well plates (2000 cells/well) and exposed to PTX and CUR3 or CUR1-12V for 72 h. Cells were subsequently subjected to SRB assay as described in the previous section. Combination index (CI-value) was calculated and used to define the nature of drug interaction (synergism if CI-value < 0.8 as; antagonism if CI-value > 1.2; and additive if CI-value ranges from 0.8–1.2).

CI-value was calculated from the formula:

$$CI-value=\frac{IC_{50}ofdrug\left(x\right)combination}{IC_{50}ofdrug\left(x\right)alone}+\frac{IC_{50}ofdrug\left(y\right)combination}{IC_{50}ofdrug\left(y\right)alone}$$

#### Statistical analysis

Data are presented as mean ± SEM using GraphPad prism^™^ software (GraphPad software Inc., La Jolla, CA, USA) for windows version 5.00. Analysis of variance (ANOVA) with Tukey’s post hoc test was used for testing the significance using SPSS^®^ for windows, version 17.0.0. p<0.05 was taken as a cut off value for significance.

## Supporting Information

S1 File Cytotoxicity dose-response curves. (DOCX)

S2 File Spectra of synthesized compounds. (DOCX)
