# Supplementary material for: Molecular Mimics of Classic P-Glycoprotein Inhibitors as Multidrug Resistance Suppressors and Their Synergistic Effect on Paclitaxel
Source: PLoS One. 2017 Jan 9;12(1):e0168938. doi: 10.1371/journal.pone.0168938 (PMC5222621; doi:10.1371/journal.pone.0168938)
Supplement: S1 File — (DOCX) [file pone.0168938.s001.docx]

# S1 File. Cytotoxicity Dose Response Curves


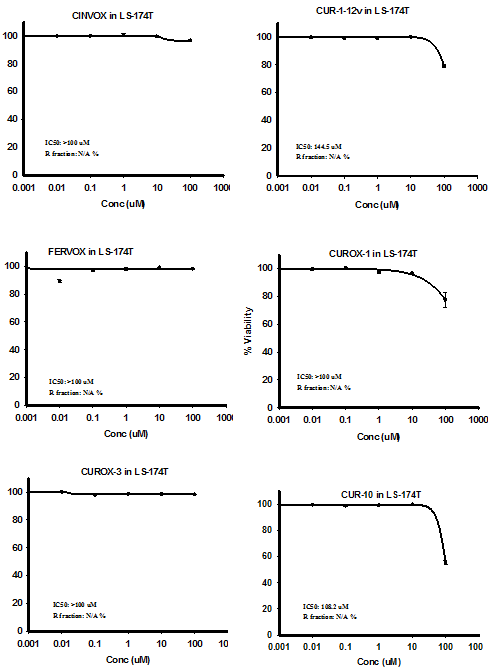


Figure A. Dose response curves of non-cytotoxic (IC_50_>100 µM) for synthesized compounds against LS-174T CRC cancer cell line. The X-axis if for concentration in µM and Y-axis is for cell viability%.


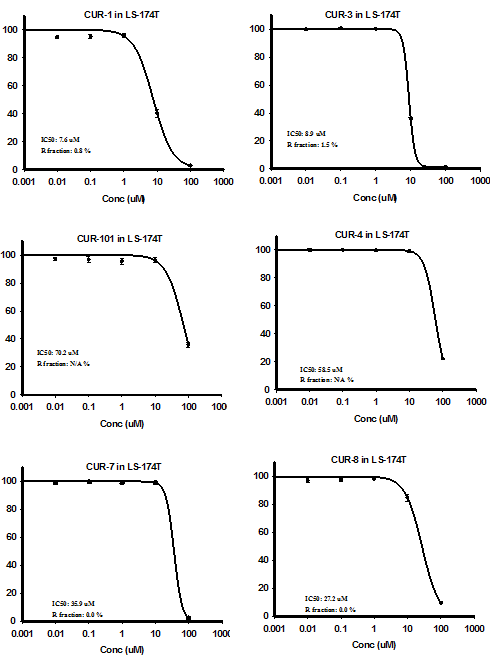


Figure B. Dose response curves of synthesized compounds with significant cytotoxic activities against LS-174T colon cancer cell line. The X-axis if for concentration in µM and Y-axis is for cell viability%.
