# Supplementary material for: Molecular Mimics of Classic P-Glycoprotein Inhibitors as Multidrug Resistance Suppressors and Their Synergistic Effect on Paclitaxel
Source: PLoS One. 2017 Jan 9;12(1):e0168938. doi: 10.1371/journal.pone.0168938 (PMC5222621; doi:10.1371/journal.pone.0168938)
Supplement: S2 File — (DOCX) [file pone.0168938.s002.docx]

# S2 File. Spectra of Synthesized Compounds


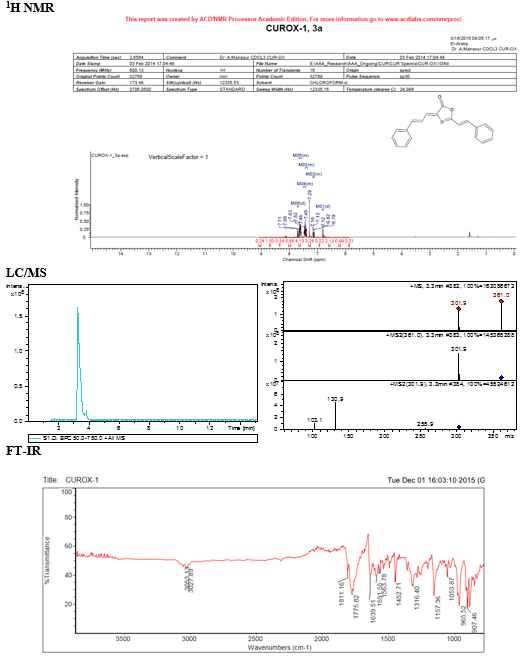


Figure A. Spectra of compound 3a (Curox-1)


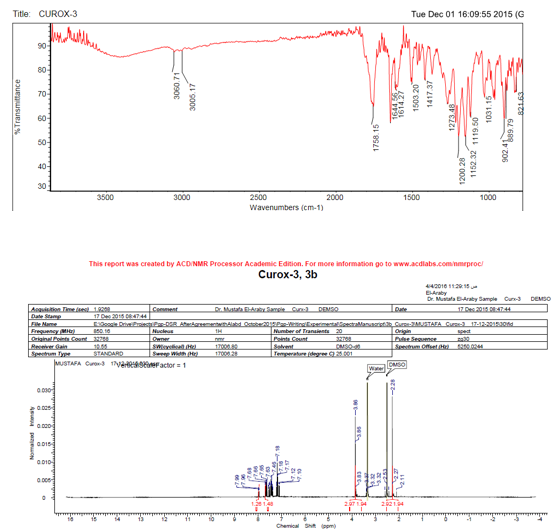


Figure B. Spectra of compound 3b (Curox-3)

##
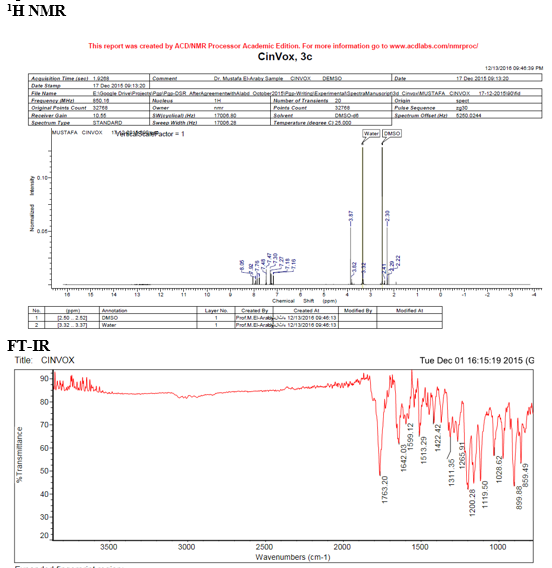


Figure C. Spectra of compound 3c (Cinvox-3)


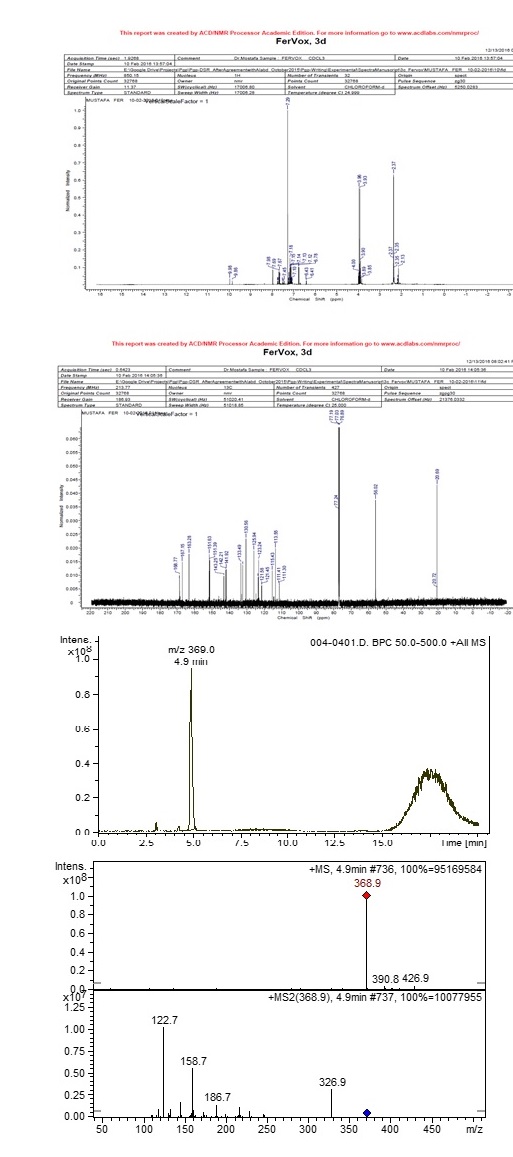


Figure D. Spectra of compound 3d (Fervox)

##
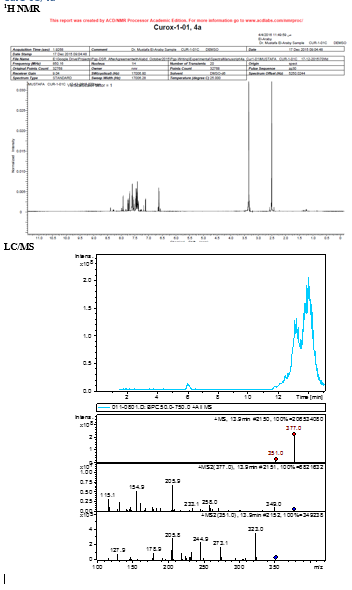


Figure E. Spectra of compound 4a (Cur1-01)


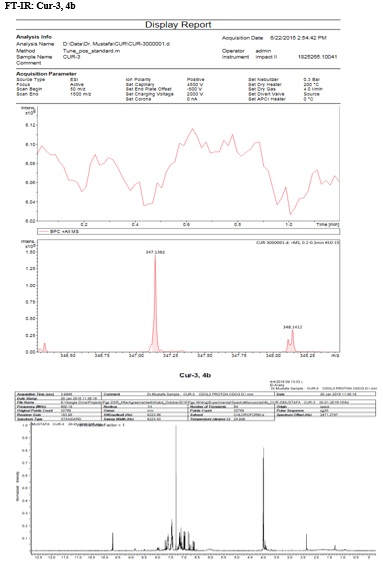


Figure F. Spectra of compound 4b (Cur-3)


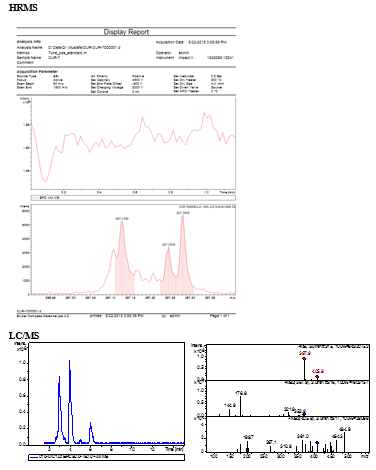


Figure G. Spectra of compound 4c (Cur-7)


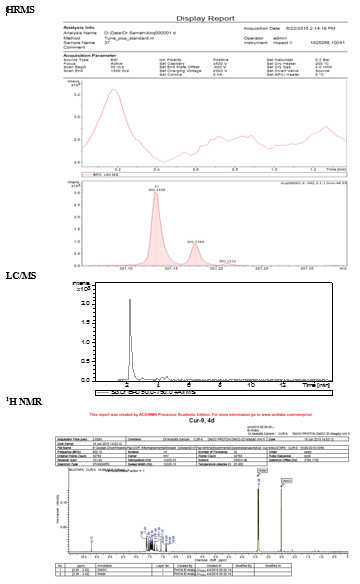


Figure H. Spectra of compound 4d (Cur-9)


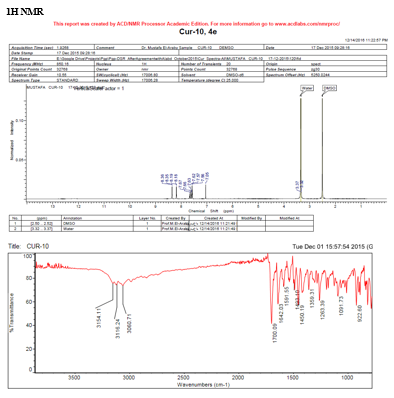


Figure I. Spectra of compound 4e (Cur-10)


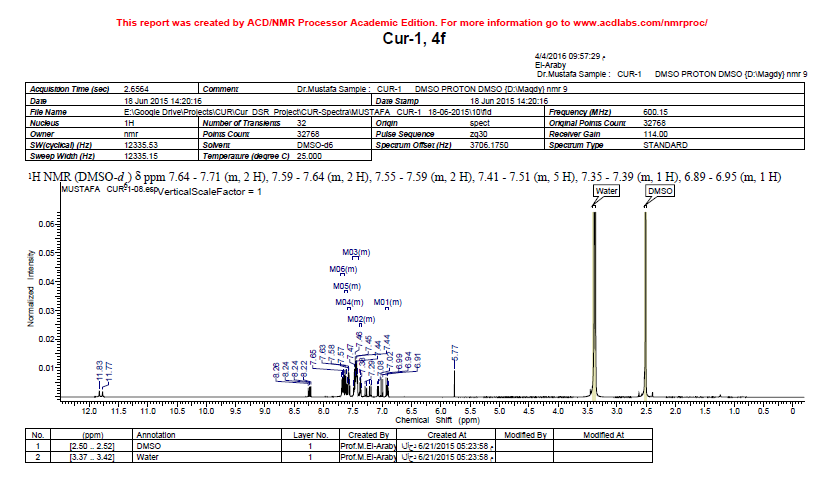


Figure J. Spectra of compound 4f (Cur-1)


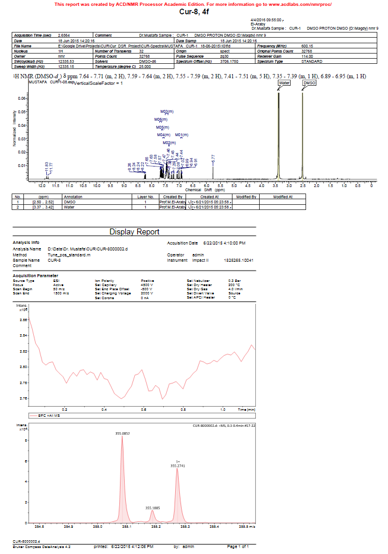


Figure K. Spectra of compound 4g (Cur-8)


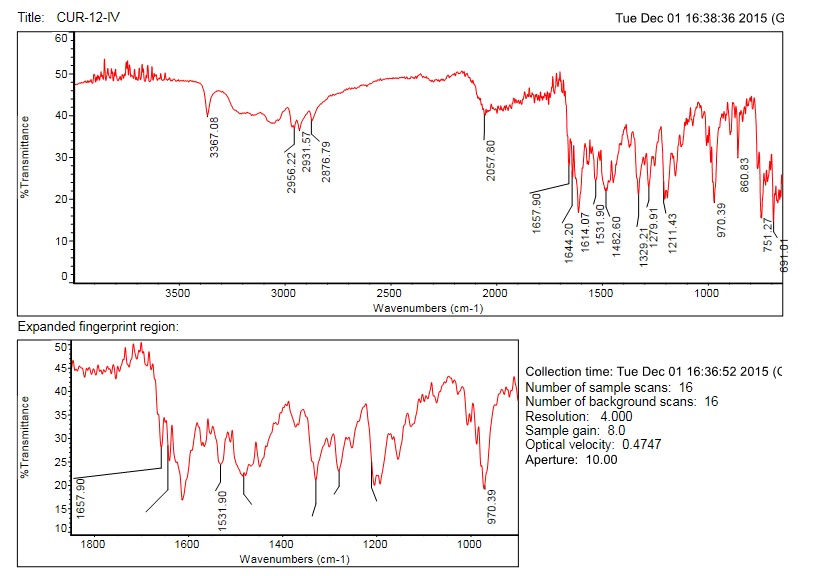


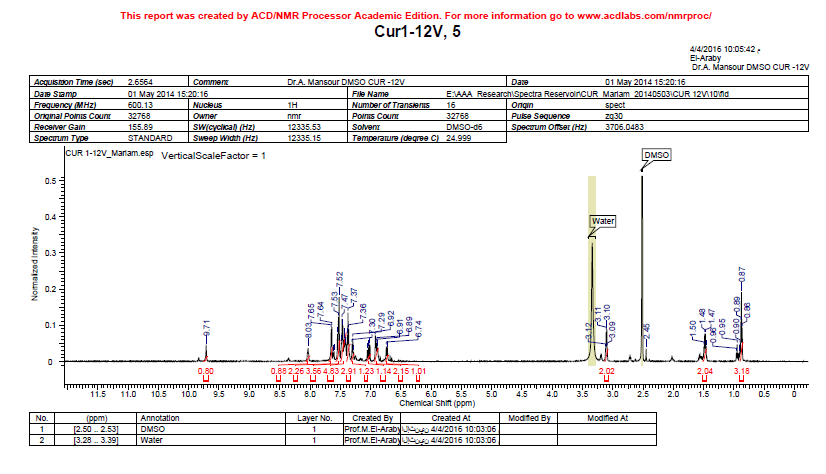


Figure L. Spectra of compound 5 (Cur-1-12V)
